# Neuroimaging Findings in Congenital Biotinidase Deficiency: A Case Report

**DOI:** 10.7759/cureus.90218

**Published:** 2025-08-16

**Authors:** Alamelu Alagappan, Amit Satapathy, Suprava Naik, Biswajit Sahoo, Pradosh Kumar Sarangi, Manoj Kumar Nayak

**Affiliations:** 1 Radiodiagnosis, All India Institute of Medical Sciences, Bhubaneswar, Bhubaneswar, IND; 2 Pediatrics, All India Institute of Medical Sciences, Bhubaneswar, Bhubaneswar, IND; 3 Radiodiagnosis, All India Institute of Medical Sciences, Deoghar, Rampur, IND

**Keywords:** acidosis, alopecia, biotinidase deficiency, holocarboxylase synthetase deficiency, supplemental biotin, symmetric diffusion restriction

## Abstract

Congenital biotinidase deficiency (CBD) is an uncommon inborn error of metabolism (IEM) that frequently manifests in infancy. Clinical manifestations vary from alopecia rash to severe neurological features like hypotonia, seizures, and developmental delay. Typical imaging manifestations will have symmetric restricted diffusion in the corona radiata, perirolandic white matter, posterior limb of the internal capsule (PLIC), parieto-occipital grey matter, brainstem, middle cerebellar peduncle (MCP), and splenium of the corpus callosum. Early diagnosis is essential because early treatment may reduce or prevent the severity of this disease. Herein, we report a case of CBD presented with breathlessness, alopecia, and hearing loss with typical imaging features, reversed with biotin supplementation in a six-year-old male child.

## Introduction

Congenital biotinidase deficiency (CBD) is an uncommon inborn error of metabolism (IEM) that often manifests in infancy. An estimated 1 in 40,000 to 1 in 60,000 babies are born with biotinidase deficiency [1]. Minor cutaneous manifestations like alopecia and rash, and severe neurological features like hypotonia, seizures, and developmental delay are major manifestations. Clinical manifestations of biotinidase deficiency vary, impacting the ophthalmologic, neurologic, dermatologic, and immune systems. Profound biotinidase deficiency typically manifests symptoms between the ages of one week and ten years. They may also exhibit signs and symptoms of numerous other inborn metabolic abnormalities like organic academia, including isolated carboxylase deficiency, dietary biotin deficiency, and holocarboxylase synthetase deficiency. Early detection is essential because prompt treatment may prevent or reduce clinical insult. Hence, magnetic resonance imaging (MRI) is indispensable for the diagnosis, followed by a biochemical assay for confirmation [2]. Herein, we report a case of a six-year-old male child who presented with breathlessness and was later diagnosed to have congenital biotinidase deficiency.

## Case presentation

**Figure 1:**
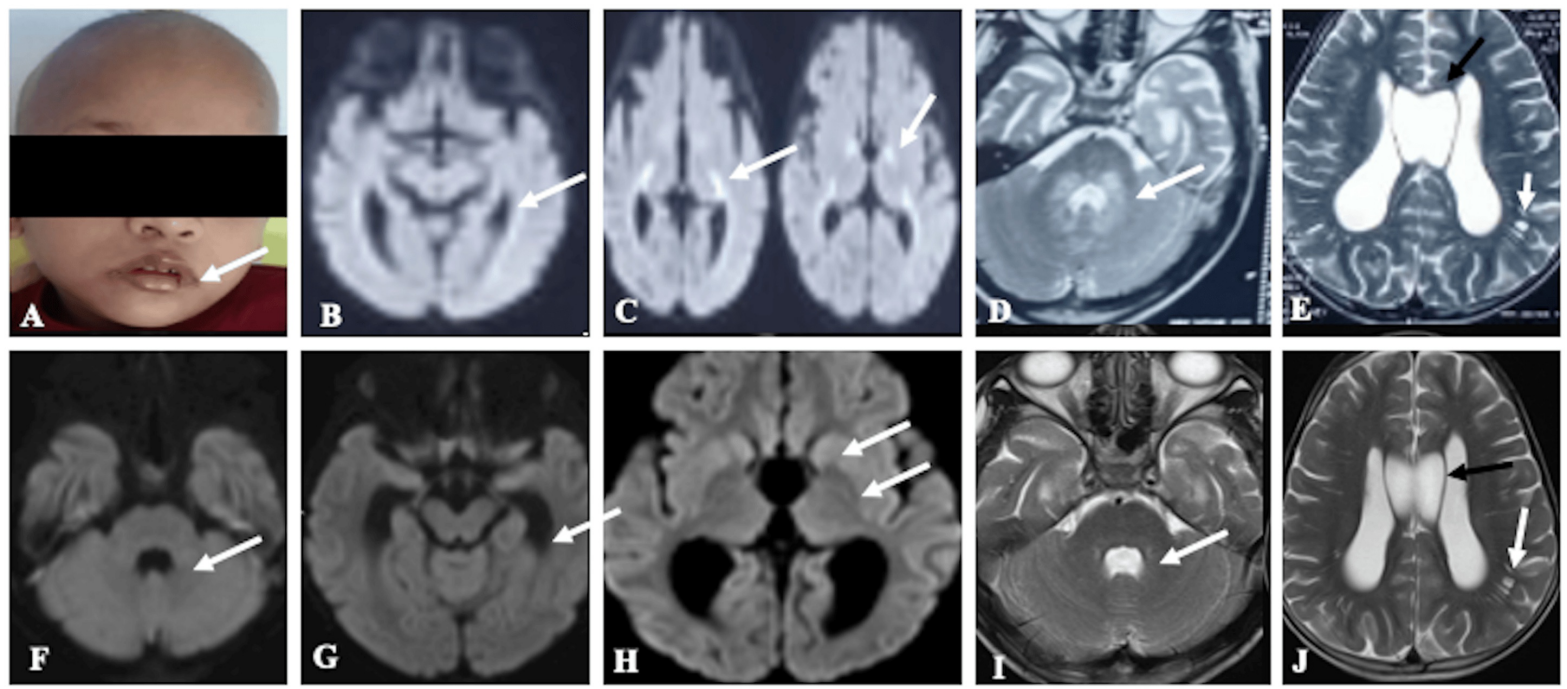
Clinical and magnetic resonance imaging (MRI) image of the patient with biotinidase deficiency Clinical photograph (A) of the patient showed alopecia with cheilitis (white arrow). The MRI image (axial DWI image) (B,C) showed relatively symmetrical restricted diffusion in the bilateral peritrigonal white matter (white arrow in B), bilateral genu, and posterior limbs of internal capsules (white arrows in C). The axial T2 image at the level of the pons (D) showed T2 hyperintensity surrounding the fourth ventricle in the pons, both middle cerebellar peduncles, and the dentate nuclei (white arrow). The axial T2 image at the level of the lateral ventricle (E) showed the cavum septum pellucidum (black arrow) and dilated Virchow-Robbins space (VRS) (white arrow). Follow-up MRI axial diffusion (F-H) and axial T2 (I,J) after two weeks of biotin supplement showed complete resolution of restricted diffusion (white arrows in F-H) and T2 hyperintensities (white arrow in I) in the corresponding regions with persistent cavum septum pellucidum (black arrow in J) and dilated VRS (white arrow in J).

A six-year-old male child, born to non-consanguineous parents, presented with a one-week history of breathlessness. Breathlessness was gradual in onset and progressive in nature. The child was hospitalized outside, where he developed erythema around the eyes and perioral region (Figure 1A), which was gradually progressing, after which he was referred to our hospital. The child had multiple episodes of seizures previously since the age of 10 months and was on valproate till three years of age, and then tapered and subsequently stopped as he was seizure-free till the current presentation. No specific diagnosis was made at the hospital where he was treated. His antenatal and perinatal history was uneventful. Family history was noncontributory. His developmental milestones were appropriate for his age. Other past histories include brainstem evoked response audiometry (BERA) screening, which was positive for bilateral sensorineural hearing loss, hair loss for the past three months, and difficulty in walking for the past three months. On examination, he was conscious, irritable, oriented, afebrile, and hyperventilating (respiratory rate was 45/min). The rest of the vitals were normal, with SpO2 at 97% on room air. Generalized non-scarring alopecia, periocular erythema, and stomatitis were evident on examination. His height was between the third and tenth centiles. The rest of the anthropometric measurements were within limits. Chest examination was unremarkable. CNS and eye examinations were normal, with no meningeal or cerebellar signs. Initially, the possibility of an inborn error of metabolism-such as biotinidase deficiency, holocarboxylase synthetase deficiency, or a mitochondrial disorder-was considered in view of recurrent seizures with dermatological features in the form of alopecia with rashes. In the initial screening for IEM, venous blood gas analysis revealed combined respiratory alkalosis and metabolic acidosis, with a pCO₂ of 5 mmHg, HCO₃⁻ of 6 mmol/L, and lactate of 5.2 mmol/L. There was severe metabolic acidosis with CO₂ washout. Urinary ketone was positive. However, his blood sugar and electrolytes were within normal limits. Serum ammonia and creatinine phosphokinase levels were normal (Table 1). He was started on bicarbonate correction following the resolution of metabolic derangement. The breathlessness was secondary to acidotic breathing, which resolved after starting bicarbonate correction. He was started on a low-protein diet with biotin supplements orally at a dose of 10 mg/day, considering the possibility of treatable conditions like biotinidase deficiency. Cheilitis also showed improvement with biotin supplementation and multivitamins. Venous blood gas parameters normalized after initiation of treatment, and the patient improved symptomatically; therefore, repeat serum biotinidase activity testing was not performed. Magnetic resonance imaging (MRI), performed at an outside center, was reviewed in a multidisciplinary committee. The images revealed areas of restricted diffusion involving the genu and posterior limbs of both internal capsules, periventricular white matter adjacent to the lateral ventricles, and T2-weighted hyperintensities surrounding the fourth ventricle, with involvement of the dorsal pons, bilateral middle cerebellar peduncles, and dentate nuclei (Figures 1B-1E). Dilated Virchow-Robin spaces (VRS) and a cavum septum pellucidum (CSP) were also noted (Figure 1E). Based on the imaging and clinical presentation, biotinidase deficiency and holocarboxylase synthetase deficiency were considered the primary differential diagnoses.

**Table 1:** Laboratory parameters observed in the patient and their normal ranges. HCO₃⁻: bicarbonate; pO₂: partial pressure of oxygen; pCO₂: partial pressure of carbon dioxide. Blood gas values are based on venous blood gas analysis.

Laboratory Parameter	Observed Value	Reference Range
pH	7.25	7.35 – 7.45
pCO₂ (mmHg)	5	35 – 45
pO₂ (mmHg)	80	70 – 100
HCO₃⁻ (mmol/L)	6	22 – 26
Lactate (mmol/L)	5	0.5 – 2.0
Urinary ketone (dipstick)	++ (positive)	negative
Glucose (mg/dL)	100	70 – 140
Serum sodium (mmol/L)	139	135 – 145
Serum potassium (mmol/L)	4.5	3.5 – 5.5
Serum chloride (mmol/L)	100	96 – 108
Serum ammonia (µmol/L)	35	15 – 45
Creatine phosphokinase (CPK) (U/L)	120	30 – 200
Serum biotinidase activity (nmol/min/mL)	0.01	4.4 – 10
Serum calcium total (mg/dL)	10.14	8.5 – 10.5
Serum phosphate (mg/dL)	5.95	4 – 7
C-reactive protein (mg/L)	1.66	< 10

Follow-up MRI performed two weeks later demonstrated complete resolution of the previously noted diffusion restriction (Figures 1F-1H) and minimal residual T2 hyperintensities around the fourth ventricle, with persistent dilated VRS and CSP (Figures 1I, 1J). Tandem mass spectrometry confirmed biotinidase deficiency, with serum biotinidase activity measuring less than 10% of normal (0.01 nmol/min/mL). Clinical exome sequencing and mitochondrial genome analysis were not pursued due to financial constraints. The child was discharged with appropriate dietary modifications, including a protein-restricted diet and supplementation with biotin, carnitine, pyridoxine, and oral bicarbonate. Use of a hearing aid was advised, along with regular clinical follow-up.

## Discussion

Around 40 years ago, research on biotin's use in curing carboxylase deficits began. Clinical responses to biotin supplementation were seen in patients with beta-methylcrotonylglycinuria, a carboxylase deficiency, in 1971 [3]. A decade later, Wolf et al. discovered biotin insufficiency-related multiple carboxylase deficiency [4]. Biotinidase (BTD) gene mutations (located at chromosome 3p25.1) cause biotinidase deficiency. The most common pattern of inheritance is autosomal recessive [1].

CBD is an uncommon form of metabolic encephalopathy where the lack of biotinidase enzyme prevents the cleavage of biotin from biocytin. It functions as a coenzyme for four carboxylases, which play an important role in amino acid metabolism, gluconeogenesis, and fatty acid synthesis [5].

CBD can be profound (<10% of mean normal enzyme activity) or partial (10 to 30%). The age of presentation greatly depends on the level of enzyme activity, with profound cases typically manifesting between six months of life and 10 years. Globally, the incidence of biotinidase deficiency ranges from 1 in 40,000 to 1 in 60,000 births, making it an uncommon disorder. In the US, partial cases ranged from 1 per 31,000 to 1 per 40,000 in 2006, while profound cases were 1:80,000 [6].

Developmental delay, seizures, ataxia, hypotonia, visual impairment, sensorineural hearing loss, skin pigmentation, alopecia, and dermatitis are a few of the clinical characteristics of CBD. Neurological symptoms like seizures, hypotonia, ataxia, and developmental delay occur in early infancy. Dermatological and metabolic manifestations are reported to occur in later stages of life, depending on the severity of deficiency. Our patient was a six-year-old child with breathlessness and a history of seizures. The patient also developed respiratory alkalosis with metabolic acidosis. These manifestations can be attributed to a lack of biotin, leading to energy loss and anaerobic metabolism, thereby increasing lactate levels. Early diagnosis with a biotin supplement in infancy can potentially reverse the underlying acid-base balance abnormalities completely.

Only a few earlier case studies have described the neuroimaging findings in CBD, which are highly dependent on the age of presentation. MRI is the imaging modality of choice. Cerebral atrophy is the most common imaging abnormality described in previous studies, which can occasionally be linked to subdural hygromas or hemorrhages. Other frequent MRI abnormalities include white matter swelling and delayed myelination [7]. The corona radiata, perirolandic white matter, posterior limb of internal capsule (PLIC), parieto-occipital grey matter, brainstem, middle cerebellar peduncle (MCP), and splenium of corpus callosum showed bilateral symmetrical diffusion restriction in diffusion imaging in four patients [7-9].

In our case, cerebral atrophy was not present. However, restricted diffusion was seen in the genu, posterior limb of the internal capsule, and periventricular white matter. A post-biotin supplement scan showed complete resolution of restricted diffusion in these areas. Magnetic resonance spectroscopy (MRS) typically exhibits a low N-acetyl aspartate (NAA), lactate peak, and reversal of the choline-to-creatine ratio [8]. NAA is considered a marker of neuronal integrity. Reduced NAA levels in biotinidase deficiency suggest potential neuronal damage or dysfunction.

Several theories have been put forth for diffuse white matter signal abnormality in infants with biotinidase deficiency, including leukodystrophy, interstitial edema, delayed myelination, dysmyelination, and ischemia. We agree with Biswas et al. that interstitial edema, neither leukodystrophy nor dysmyelination, is the primary process causing these alterations [10]. Our patient and the majority of patients in their series had a diffuse pattern of involvement, and these changes were reversible, which supports this hypothesis.

The major differential diagnosis in our case was holocarboxylase deficiency. Neurological and dermatological manifestations in both biotinidase deficiency and holocarboxylase deficiency are quite similar. This similarity can be explained by the fact that holocarboxylase synthetase deficiency results from a defect in the enzyme responsible for attaching biotin to carboxylases. Hence, differentiating the two based solely on clinical features is difficult. MRI findings in holocarboxylase synthetase deficiency may include subependymal cysts, ventriculomegaly, and intraventricular hemorrhage. Other major findings are diffuse cerebral edema, cerebral atrophy, and diffusion restriction in the cerebellum and bilateral basal ganglia [11]. Seizures with encephalopathy and acidosis can also be features of other organic acidemias. In infants, seizures and altered sensorium are often initially attributed to sepsis, which is far more commonly encountered in day-to-day clinical practice. Although neurological manifestations in biotinidase deficiency can mimic those of Maple Syrup Urine Disease (MSUD), the characteristic burnt sugar or maple syrup odor of urine, feeding difficulties, and the MRI findings in our case helped in ruling out MSUD as a differential diagnosis [12].

The degree of biotinidase deficiency (less than 10% vs. 10-30% of mean normal enzyme activity) and its impact on the treatment strategy are important considerations. In other circumstances, there may be no or few symptoms. Treatment with biotin usually reverses most of the symptoms, except in cases of moderate-severe developmental delay, optic atrophy, and established hearing loss. Similarly, early biotin supplementation can prevent the development of clinical manifestations of biotinidase deficiency. Hence, many countries are now doing routine neonatal screening as a protocol for biotinidase deficiency [13].

The recommended treatment of biotin replacement is 5 to 20 mg daily, which can be increased up to 40 mg per day if the symptoms persist. Apart from replacement therapy, it is important to counsel the family members regarding the lifelong need for supplementation. As the disorder is autosomal recessive, parents must explain that there is a 25% probability with each pregnancy of having another child with this condition [13].

## Conclusions

The clinical characteristics of biotinidase deficiency may mimic other IEMs. Neurological and dermatological manifestations, apart from the characteristic MRI features and MRS findings like elevated lactate peaks, as reported in the present case and a few other prior written case reports or case series, will help in the early diagnosis of this disease. Medical geneticists play a vital role in confirming the diagnosis. A multidisciplinary approach may aid early diagnosis and treatment, preventing irreversible neurological damage and complications.
